# Kansas and Missouri Valley Dental Association

**Published:** 1872-08

**Authors:** Junius E. Cravens

**Affiliations:** Kansas City, Mo.


### Kansas and Missouri Valley Dental Association.

KANSAS CITY, MO., July 19, 1872.

DR. J. TAFT—*Dear Sir:*

In response to your favor of the 16th inst. asking synopsis of organization, the membership, officers, &c., of the Kansas and Missouri Valley Dental Association, I send you herein a few items, taken from the society records.

At a meeting of the dentists of Kansas City and vicinity, held January 17, 1870, at the office of Dr. Prevost (city), for the purpose of perfecting the organization of a Dental Association, the following business was transacted, Dr. Hammond in the chair: Adopted constitution and by-laws of the Hudson Valley Dental Association (subject to revision); adopted name of the Kansas and Missouri Valley Dental Association. Officers elected for the ensuing year, G. W. Tindall, President; Wm. M. Hammond, Vice President; Sam. B. Prevost, Secretary; J. K. Stark, Treasurer; Executive Committee, C. Prevost, H. J. Traver.

The society adjourned, holding special meetings a number of times after organization up to March 21, 1870. The total membership at that time was about eight, some of whom lived outside of Kansas City, and could not attend promptly at all meetings- The interest on the part of some members began to flag, and the others having become discouraged, the society relapsed into a peaceful slumber, from which it was aroused by mutterings of hostility, and held a called meeting on April 19, 1872, at the (city) residence of Dr. Tindall, President. During the two years of comitose sleep, the number of local dentists had become augmented some, and the called meeting of the society added several new names to its roll; at other subsequent meetings new names were added, until the present membership amounts to fourteen, two residing in other towns. I said our number was fourteen, but I wish to correct that by calling attention to the expulsion of H. J. Traver, on Tuesday, the 2nd inst., for misdemeanors which are explained in a report which I have already sent you for publication.

After the resurrection of the Kansas and Missouri Valley

Dental Association this spring, we adopted the ethics, constitution and by-laws of the American Dental Association, and designated the first Tuesday in each month as a day for regular official meetings, while the third Tuesday in each month is designated for special meetings, upon which occasions the exercises are entirely of the nature of essays and discussions on topics of practical interest to dentists.

We have resolved our society into a dental free dispensary, and all persons coming to the designated office, accompanied by a pauper's certificate from any recognized physician, are treated free of charge. The physicians of the city are notified each month of what dental office will render gratuitous services to worthy cases for that month, under the auspices of the Kansas and Missouri Valley Dental Association. The following is the form of notices sent to physicians.

KANSAS CITY, Mo., ——187

Dr. ———, the Kansas and Missouri Valley Dental Association having formed a free Dental Dispensary, you are requested to send any charity patients applying at your office to Dr. ———, Dentist, office at ——- street. He will attend to all such cases, if accompanied by your certificate and the case is a charity one. Dr. ———, Secretary.

I wish to say that this society stands *squarely* on the subject of ethics, and will not hesitate to expel any or all members who transgress the bounds of professional gentility. Our quorum is placed at five, including the presiding officer, and we intend, if necessary, to expunge everything like quackery until there shall be nothing but a quorum left to mark where once we stood.

The present officers of the Association are: J. K. Stark, President; S. B. Prevost, Vice President; J. E. Cravens, Cor. and Rec. Secretary; C. F. Rankin, Treasurer; John N. Tremaine and H. C. Massie, Executive Committee.

I thank you in behalf of the association for the interest your letter implies that you take in us, and close with the expressed hope that the Kansas and Missouri Valley Dental Association may thrive in virtue and wisdom and honor, if not in numbers. Yours truly, JUNIUS E. CRAVENS.

